# HMOX2-driven crosstalk between vascular aging and heart failure: A multimodal bioinformatics and explainable machine learning approach with experimental validation

**DOI:** 10.1371/journal.pone.0357886

**Published:** 2026-09-15

**Authors:** Jinze Li, Guiting Zhou, Qiaochu Wang, Zhixuan Song, Jiantao Liu, Changzao Shen, Chuanjin Luo, Dawei Wang

**Affiliations:** 1 Guangzhou University of Chinese Medicine, Guangzhou, China; 2 The First Clinical Medical College, Guangzhou University of Chinese Medicine, Guangzhou, China; 3 Shunde Hospital of Guangzhou University of Chinese Medicine, Foshan, China; 4 The First Affiliated Hospital of Guangzhou University of Chinese Medicine, Guangzhou, China; 5 State Key Laboratory of Traditional Chinese Medicine Syndrome, Guangdong, China; Augusta University, TAIWAN

## Abstract

**Purpose:**

The molecular mechanisms linking vascular aging (VA) and heart failure (HF) remain elusive, hindering therapeutic strategies for their comorbidity. This study aimed to identify key biomarkers and pathways potentially involved in VA-HF synergy using integrative computational approaches.

**Methods:**

We analyzed the GSE57338 dataset (136 controls, 177 HF samples) to identify HF-associated differentially expressed genes (DEGs) and constructed a weighted gene co-expression network (WGCNA). Vascular aging-related targets were retrieved from GeneCards (n = 16,243). Consensus genes (CGs) were derived by intersecting DEGs, WGCNA hub genes, and VA-related targets. Functional enrichment, machine learning prioritization (LASSO regression, Random Forest, and SHAP-XGBoost), and network analysis (GeneMANIA) were applied to identify potential regulators. Top candidates were experimentally validated using qPCR in doxorubicin-induced rat primary vascular smooth muscle cells and human VSMC cell line for the VA model, and H9C2 cardiomyoblast injury model for HF. Further validation was performed in a mouse model of doxorubicin-induced HF, assessing cardiac function by echocardiography, myocardial fibrosis by Masson’s trichrome staining, and vascular aging markers (P16, P21) by qPCR.

**Results:**

We identified 272 CGs enriched in cGMP-PKG signaling, cytoskeletal regulation, and PPAR pathways. Machine learning prioritized 12 core genes, with HMOX2 as the top predictor (AUC = 0.978). qPCR analysis confirmed upregulation of HMOX2, S1PR3, and SERPINA3 in doxorubicin-treated rat primary vascular smooth muscle cells, human VSMC cell line, and H9C2 cardiomyoblasts compared to controls (*P* < 0.05). In the mouse model, doxorubicin administration induced significant cardiac dysfunction and myocardial fibrosis, accompanied by elevated expression of senescence markers P16 and P21 in vascular tissues. These findings collectively suggest that these genes may play important roles in VA-HF comorbidity (*P* < 0.05).

## Introduction

Heart failure (HF) remains a global health crisis, affecting over 64 million individuals worldwide, with escalating morbidity and mortality despite advances in therapeutic strategies [1,2]. Emerging evidence highlights vascular aging (VA), a pathological process characterized by endothelial dysfunction, arterial stiffness, and chronic inflammation, as a contributor to HF progression [3,4]. Vascular aging is defined as the progressive degenerative changes in the morphology and function of blood vessels that occur with increasing age. Morphologically, it is characterized by increased deposition of collagen fibers, fragmentation and disorganized arrangement of elastic fibers, disordered alignment of smooth muscle cells, and intimal thickening, leading to vessel wall thickening, luminal dilation or stenosis, and reduced elasticity. Functionally, vascular aging manifests as increased arterial stiffness, decreased sensitivity to vasodilatory factors, increased sensitivity to vasoconstrictive factors, impaired angiogenesis, endothelial dysfunction, and elevated secretion of inflammatory cytokines. In brief, vascular aging is a physiological process in which blood vessels progressively degenerate in both structure and function, lose normal elasticity and regulatory capacity, and consequently increase the risk of cardiovascular disease [5]. The interplay between VA and myocardial remodeling involves complex molecular crosstalk, yet the shared genetic drivers and mechanistic pathways underlying this relationship remain poorly elucidated [3]. Traditional biomarker discovery approaches often fail to capture the systemic complexity of HF-VA interactions, underscoring the need for integrative frameworks that combine multi-omics data with advanced computational models [6].

Recent studies have identified aging-related vascular dysfunction as a precursor to cardiac decompensation, mediated through oxidative stress, impaired angiogenesis, and cytoskeletal dysregulation [7,8]. However, systematic identification of hub genes bridging these processes remains limited. Furthermore, while machine learning (ML) has facilitated biomarker prioritization, its application in deciphering VA-HF networks, particularly using interpretable AI tools, is still developing [6,9,10]. Here, we combined differential expression analysis, co-expression network analysis, and explainable machine learning to unravel the molecular links between VA and HF, followed by experimental validation. In this study, we integrated bioinformatics, machine learning, and experimental validation to systematically investigate the molecular crosstalk between VA and HF.

## Methods

### Biomarker collection for “heart failure” and “vascular aging” and deg Analysis

The raw expression profile datasets of HF patients were downloaded from the GEO database (https://www.ncbi.nlm.nih.gov/geo/), specifically the GSE57338 dataset (platform: GPL11532), which includes 136 healthy arterial tissue samples and 177 HF tissue samples. Vascular aging-related biomarkers were retrieved from the GeneCard database, yielding a total of 16,243 potential biomarkers. DEG analysis on the GSE57338 dataset was conducted using the “limma” package in R software. The cutoff criteria for identifying DEGs were set at a FDR (adj.P.Val) < 0.05 and |logFC| > 0.5, ensuring stringent selection of differentially expressed genes under these conditions.

### WGCNA and identification of key module genes

The “WGCNA” package was utilized to construct a scale-free co-expression gene network for the GSE57338 dataset. The median absolute deviation (MAD) was calculated for each gene across the datasets, and the top 5,000 genes with the highest MAD were selected for further analysis. The “goodSamplesGenes” function was employed to identify missing entries and entries with weights below the threshold, as well as genes with zero variance in the data. This function returned lists of samples and genes that met the criteria for maximum missing values or low weight values. Additionally, the correlation between module eigengenes and sample trait matrices was computed. These correlations, along with their corresponding p-values, were visualized using the “labeledHeatmap” function. Modules showing the strongest correlations with traits were selected for further analysis [11].

### Functional enrichment analysis

To identify overlapping genes among the differentially expressed gene sets, a Venn diagram analysis was performed using the online tool jvenn (https://jvenn.toulouse.inrae.fr/app/example.html), resulting in the identification of Consensus genes (CGs). These CGs were subsequently subjected to Gene Ontology (GO) and Kyoto Encyclopedia of Genes and Genomes (KEGG) pathway enrichment analyses using a series of R packages, including org.Hs.e.g.,db, GOplot, enrichplot, and clusterProfiler. Enrichment results with a *P*-value < 0.05 were considered statistically significant. Visualization was performed using ggplot2, circlize, RColorBrewer, and ComplexHeatmap to present enriched functions and pathways through circular plots, bar charts, and heatmaps.

### LASSO regression and random forest analysis

To further refine the selection of potential biomarkers associated with vascular aging combined with heart failure, LASSO (Least Absolute Shrinkage and Selection Operator) regression and Random Forest (RF) analysis were employed. LASSO regression was conducted using the “glmnet” package to perform feature selection on the 272 CGs. This method applies L1 regularization to shrink the coefficients of less relevant features to zero, thereby identifying key predictors. In parallel, the RF algorithm was implemented via the “randomForest” package, which ranked gene predictors based on their contribution to classification accuracy. These variable importance scores were used to narrow the list of candidate biomarkers.

### GeneMANIA database analysis

To explore the interaction networks of co-expressed differentially expressed genes associated with vascular aging and heart failure, we utilized the GeneMANIA database (http://genemania.org/) for the analysis of 12 core genes. This involved constructing interaction networks based on co-expression, physical interactions, shared protein domains, and predicted associations. The “assign based on query GeneMANIA” strategy was employed to maximize connectivity among all input genes. This approach uses linear regression to automatically assign weights, aiming to maximize interactions within the list of genes while minimizing connections with genes not included in the list. This ensures that the resulting network highlights the most relevant interactions pertaining to the queried genes, thereby enhancing the understanding of their functional relationships and potential roles in vascular aging and heart failure. This network supported interpretation of functional relationships and potential roles of these genes in vascular aging and heart failure.

### Machine learning framework and SHAP interpretability

The analytical strategy employed XGBoost (eXtreme Gradient Boosting), a high-performance ensemble learning algorithm built on gradient-boosted decision trees. XGBoost enhances prediction accuracy through an iterative training process, in which each new tree is trained to correct the errors of previously built trees. The algorithm also integrates L1 (Lasso) and L2 (Ridge) regularization techniques to minimize overfitting and improve model generalization. Recognized for its computational efficiency and strong predictive capability, XGBoost has been widely adopted in clinical and epidemiological research for both classification and regression problems.

To improve transparency and interpretability of the model, SHAP (SHapley Additive exPlanations) values were computed using concepts from cooperative game theory. This mathematically rigorous method measures the contribution of each input feature to individual predictions by evaluating its average marginal effect across all possible feature combinations. SHAP not only reveals overall feature importance but also provides detailed, case-specific explanations for model outputs. By combining the predictive power of XGBoost with the explainability offered by SHAP, we established a framework suitable for supporting clinical decision-making.

### Experiment model establishment

#### Animal model.

All animal experiments in this study were conducted in accordance with international ethical guidelines for animal research and were approved by the Experimental Animal Ethics Committee of Guangzhou University of Chinese Medicine (No. 20240520003). To investigate the mechanisms of heart failure and vascular aging, 8-week-old male C57BL/6J mice were randomly divided into a model group and a control group. The model group received intravenous injections of doxorubicin (5 mg/kg) via the tail vein once a week for 6 consecutive weeks to induce chronic injury, while the control group received equivalent volumes of normal saline on the same schedule. During the entire modeling period, mice were monitored twice daily (at 9:00 AM and 5:00 PM) for general health status, including body weight changes, locomotor activity, fur condition, respiratory rate, and food/water intake. No deaths occurred in either group throughout the experiment. All observations were independently recorded by two trained experimenters and cross-verified. After all in vivo functional assessments were completed, the mice were deeply anesthetized by an intraperitoneal overdose of sodium pentobarbital, followed by cervical dislocation for euthanasia to collect cardiac and vascular tissues for subsequent analysis.

#### In vitro vascular aging model.

Both rat primary vascular smooth muscle cells and the Human Aortic Smooth Muscle Cells line (HASMC) in the logarithmic growth phase were digested, counted, and seeded into 6-well plates at a density of 1 × 10^5^ cells per well. To induce cellular senescence, the model group was treated with culture medium containing 1.0 μM doxorubicin for 24 hours. Control group cells received an equivalent volume of phosphate-buffered saline (PBS) instead of doxorubicin. Following incubation, the medium in all wells was carefully aspirated, and cells were washed once with PBS. Fresh, drug-free complete medium was then added to all wells, and cultures were maintained for an additional 3 days to establish the vascular aging model. For HMOX2 knockdown experiments, cells were transfected with 50 nM HMOX2‑specific siRNA (sense, 5′‑GCGGAGACUGACUGACCUATT‑3′; antisense, 5′‑UAGGUCAGUCAGUCUCCGCTT‑3′; Sangon Biotech) or an equivalent concentration of negative control siRNA (NC) using Lipofectamine™ RNAiMAX Transfection Reagent (Thermo Fisher Scientific) according to the manufacturer’s instructions. The siRNA was designed to target the coding region of rat Hmox2 mRNA (accession number NM_001127615.1) and showed no predicted cross‑reactivity with rat Hmox1 by BLAST. Cells were incubated for 48 h prior to subsequent treatments.

#### In vitro heart failure model.

H9C2 cardiomyoblasts in the logarithmic growth phase were digested, counted, and seeded into 6-well plates at a density of 2 × 10^5^ cells per well (achieving ~70–80% confluency at the start of treatment). To model heart failure, cells in the model group were exposed to culture medium supplemented with 1.0 μM doxorubicin for 24 hours. Control group cells were treated with an equivalent volume of PBS. After the treatment period, the medium was aspirated, cells were rinsed once with PBS, and fresh, drug-free complete medium was replenished. Cultures were subsequently maintained for a further 3 days to generate the heart failure model.

### Cardiac function and histological analysis

Cardiac function was assessed by transthoracic echocardiography. Briefly, mice were anesthetized with isoflurane, and M-mode echocardiograms were obtained from the parasternal short-axis view using a high-resolution small animal ultrasound system. Left ventricular internal dimensions at end-systole and end-diastole were measured. Left ventricular ejection fraction and fractional shortening were calculated to evaluate cardiac systolic function. Following echocardiography, hearts were harvested, fixed in 4% paraformaldehyde, and embedded in paraffin. Tissue sections (5 μm thick) were stained for collagen deposition using a Masson’s Trichrome Stain Kit (Solarbio, China) according to the manufacturer’s instructions. Stained sections were imaged under a light microscope, where cardiomyocytes appeared red and collagen fibers blue. The collagen volume fraction was quantified ImageJ software.

### Reverse transcription quantitative PCR (RT-qPCR)

Total RNA was extracted from mouse vascular tissue, VSMCs, HVSMCs, and H9C2 using TRIzol reagent and Direct-zol™ RNA MiniPrep Kit. RNA was reverse-transcribed into complementary DNA (cDNA) using the PrimeScript^TM^ RT Reagent Kit, following the manufacturer’s instructions. Quantitative PCR was performed using SYBR Green chemistry on an Applied Biosystems 7900HT Fast Real-Time PCR System. Gene expression was normalized to β-actin. We used the 2-ΔΔCt method to determine the relative RNA expression. Below are the primer sequences (F: Forward; R: Reverse).

Rat:

S1PR3 (F): 5′-ACTCTCCGGGAACATTACGAT-3′.
S1PR3 (R): 5′-CCAAGACGATGAAGCTACAGG-3′.
SERPINA3 (F): 5′-TCTCCGCATGCCCAAATTCT-3′
SERPINA3 (R): 5′-GCCTTGTGCACCACTTGAGA-3′
HMOX2 (F): 5′-GCGGAGACTGACTGACCTAC-3′
HMOX2 (R): 5′-CAGAAAGGTCTGCCATTTTGGT-3′

Human:

S1PR3 (F): 5′-TGGTCAGGATCTGGACAACG-3′.
S1PR3 (R): 5′-TTGAAAAAGGGCTCCTCCGT-3′.
SERPINA3 (F): 5′-ACTCCAGACAGACGGCTTTG-3′
SERPINA3 (R): 5′-CTCTCCATTCTCAACTCTGCCT-3′
HMOX2 (F): 5′-CAGCGGAAGTGGAAACCTCA-3′
HMOX2 (R): 5′-CGAGAGGTCAGCCATTCTCA-3′

Mouse:

P16 (F): 5′-TGAATCTCCGCGAGGAAAGC-3′
P16 (R): 5′-TGCCCATCATCATCACCTGAA-3′
P21 (F): 5′-TAAGGACGTCCCACTTTGCC-3′
P21 (R): 5′-CTGAGGATCACCCCCAGGTA-3′

### SA-β-gal staining

Cellular senescence was assessed using a Senescence β-Galactosidase Staining Kit (Shanghai Biyuntian Biological Co., Ltd.). Following the model establishment period, cells were washed with PBS, fixed, and then incubated with the X-gal-containing staining solution at 37°C in a CO2-free incubator overnight. Senescent cells were identified by the presence of blue cytoplasmic staining, and the SA-β-gal positive area was quantified using image analysis software.

### Data analysis

The data, which exhibited a normal distribution, were presented as the mean ± standard deviation (SD). A comprehensive statistical analysis was conducted using the GraphPad Prism 9.0 software. The t-test or the one-way analysis of variance (ANOVA) was employed to analyze the experimental data, with a significance level of *P* < 0.05 being deemed as statistically significant.

## Results

### DEG analysis and WGCNA

**Fig 1:**
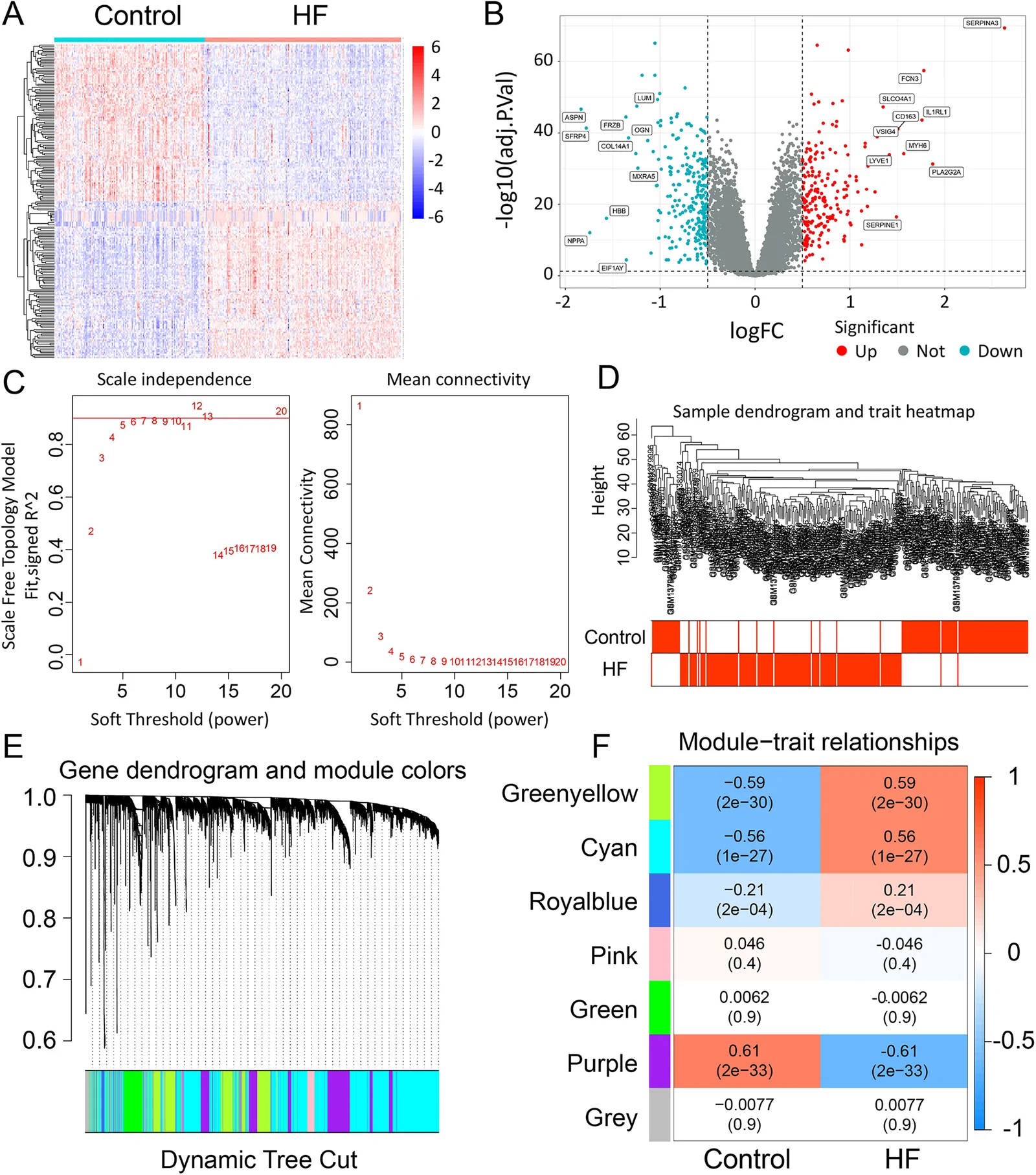
DEG and WGCNA analysis of targets in vascular aging with heart failure. A-B: Heatmap and volcano plot depicting the differential gene expression analysis between HF patients and normal controls. These visualizations highlight the genes that are significantly upregulated or downregulated. C: Analysis of scale-free topology fitting indices and mean connectivity for the GSE57338 dataset, illustrating the optimal soft-thresholding power to construct a scale-free network. D: Sample dendrogram of the GSE57338 dataset. The upper half indicates each sample ID, while the lower half denotes the group classification (HF vs. Control). E: Cluster dendrogram of the GSE57338 dataset. The upper portion displays the hierarchical clustering tree of genes, while the lower portion shows the gene modules, also referred to as network modules. F: Heatmap showing the correlation between HF phenotypes and genes in the GSE57338 dataset. The leftmost color block represents different modules, while the rightmost color bar indicates the range of correlations. In the central heatmap, deeper colors signify stronger correlations: red indicates positive correlations, and blue indicates negative correlations. Each cell includes numbers representing both the correlation coefficient and its significance level.

In the GSE57338 dataset, 450 DEGs were identified in the HF group compared to the control group, including 248 downregulated and 202 upregulated DEGs (Fig 1A, B). WGCNA was performed on the same dataset, with the optimal soft-thresholding power determined to be 10. Using this threshold, six co-expression modules were constructed (Fig 1C, D). Among these, the Purple module showed the strongest negative correlation with HF (2,669 genes, r = −0.61, *P* = 2e-33) (Fig 1E, F).

### Functional enrichment analysis of CGs

**Fig 2:**
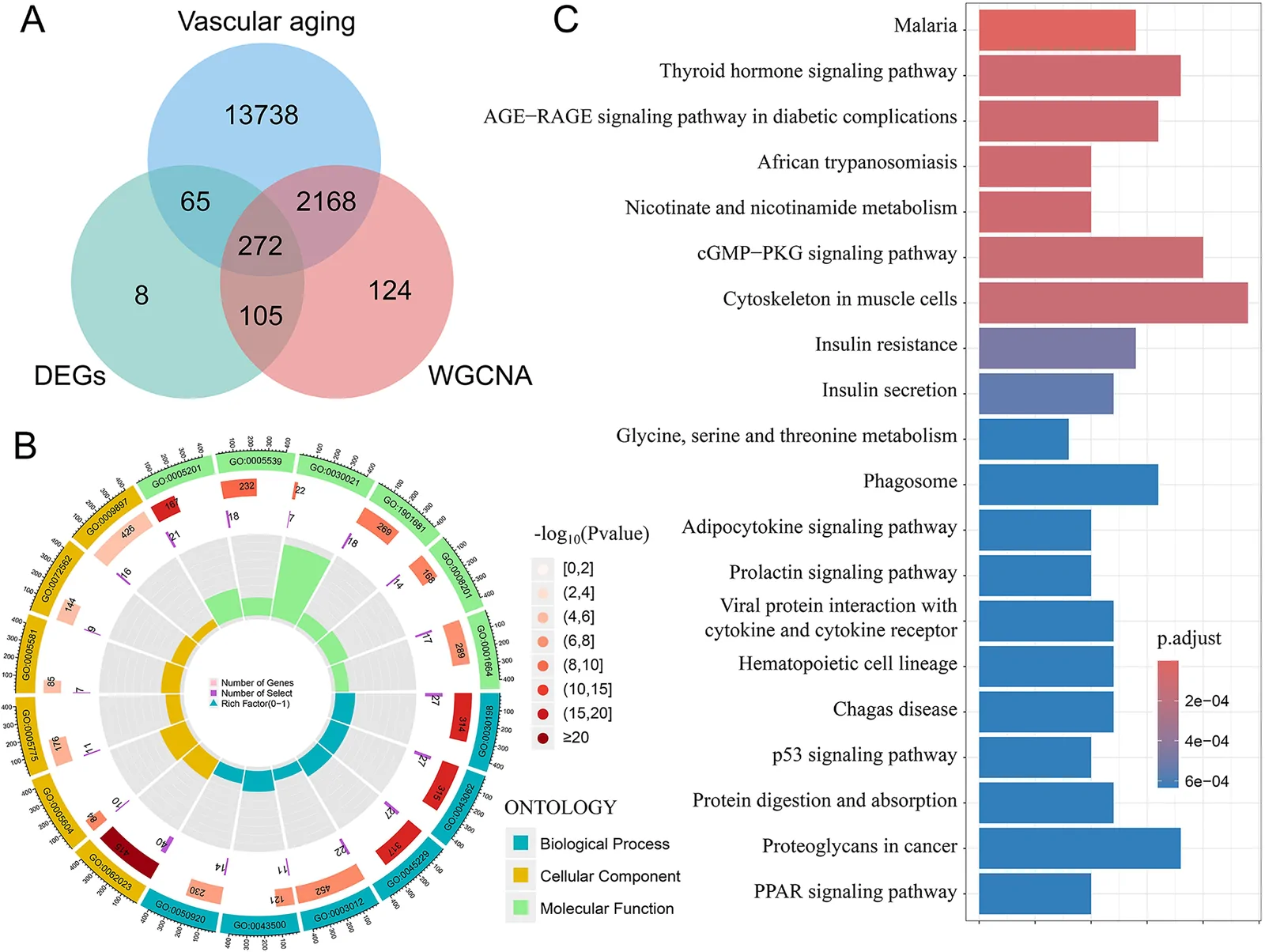
Functional enrichment analysis based on CGs. A: Venn diagram illustrating the intersection of differentially expressed genes (DEGs) from the GSE57338 dataset, key module genes identified through WGCNA, and vascular aging-related biomarkers, resulting in the identification of CGs. B: Circular plot depicting the results of Gene Ontology (GO) enrichment analysis based on the CGs. This visualization highlights the biological processes, cellular components, and molecular functions enriched within the gene set. C: Bar chart presenting the outcomes of Kyoto Encyclopedia of Genes and Genomes (KEGG) pathway enrichment analysis based on the CGs.

To identify the CGs associated with HF, we intersected DEGs from the GSE57338 dataset, key module genes derived from WGCNA, and vascular aging-related biomarkers obtained from the GeneCard database. This intersection yielded a total of 272 CGs (Fig 2A). To gain deeper insights into the functions and specific mechanisms of these CGs, functional enrichment and KEGG pathway analyses were performed. Gene Ontology (GO) analysis focusing on biological processes (BP) revealed that these CGs are predominantly enriched in pathways such as “regulation of angiogenesis,” “regulation of vasculature development,” “vascular process in circulatory system,” “regulation of blood circulation,” and “positive regulation of epithelial cell proliferation.” In terms of cellular components (CC), the GO analysis indicated that these disease-associated genes are primarily located within structures like the “sarcomere,” “myofibril,” “contractile fiber,” and “basal part of cell.” For molecular function (MF), our results highlighted significant associations with “extracellular matrix structural constituent,” “glycosaminoglycan binding,” “sulfur compound binding,” and “receptor ligand activity” among the CGs (Fig 2B). Further, KEGG pathway analysis demonstrated that these CGs are significantly involved in several critical pathways, including “Cytoskeleton in muscle cells,” “cGMP-PKG signaling pathway,” “Insulin resistance,” and “PPAR signaling pathway” (Fig 2C).

### Identification of key targets for HF with VA using LASSO regression and RF algorithms

**Fig 3:**
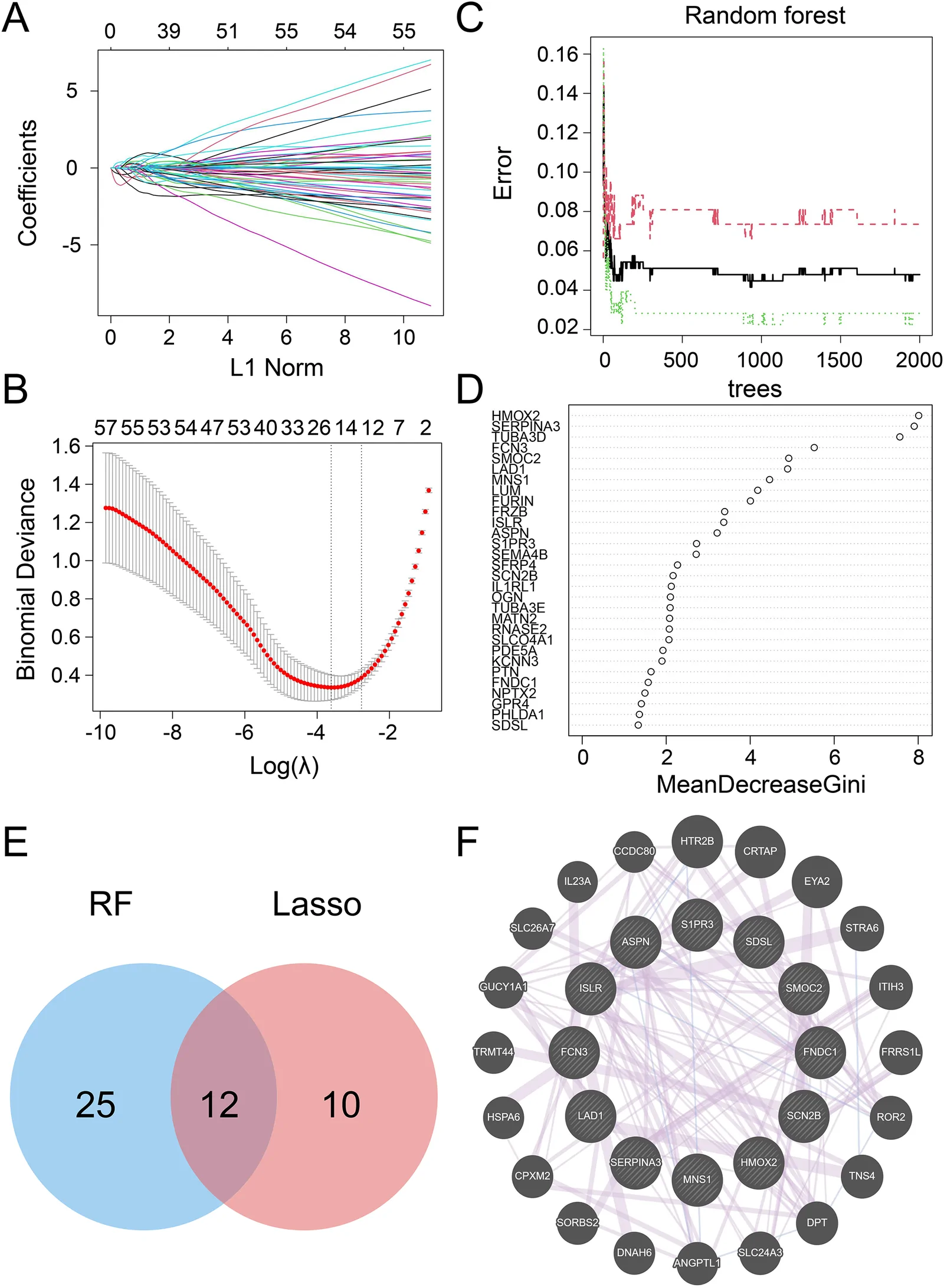
Lasso regression analysis, random forest algorithm, and geneMANIA database analysis. A-B: Lasso regression analysis was performed on 272 consensus genes (CGs) to determine the optimal lambda (λ) value for diagnostic biomarkers (A), along with the minimum lambda value that minimizes model error (B). C-D: The Random Forest algorithm was applied to the same 272 CGs to generate a Random Forest plot. Biomarkers with a MeanDecreaseGini score greater than 1 were selected for further investigation. E: A Venn diagram illustrates the intersection between the results obtained from Lasso regression and Random Forest analysis, identifying 12 Key Genes shared by both methodologies. F: These 12 key genes were further analyzed using the GeneMANIA database, revealing a network comprising 32 genes and 116 connections.

To further narrow potential targets, we applied LASSO regression and RF analysis to the 272 CGs. LASSO regression identified 22 genes (Fig 3A, B), while RF identified 37 genes contributing to classification accuracy (Fig 3C, D). Twelve core genes were obtained by intersecting the two feature sets (Fig 3E). These genes represent candidates for further exploration of shared mechanisms in HF and vascular aging.

### GeneMANIA database analysis

To uncover the potential disease-causing genes and underlying mechanisms associated with vascular aging-related heart failure, we utilized the GeneMANIA database to analyze interactions among the 12 core genes. As illustrated in Fig 3F, nodes in this network include both the uploaded genes and additional genes retrieved from the GeneMANIA database that are linked to our initial set. The lines connecting these nodes represent different types of interaction networks. Our constructed network comprises a total of 32 genes, including the original 12 uploaded genes and an additional 20 related genes, connected by 116 links. These connections encompass various types of interactions such as co-expression, physical interactions, shared protein domains, and predicted associations.

### Integrated machine learning analysis of VA and HF risk: application of XGBoost and SHAP

**Fig 4:**
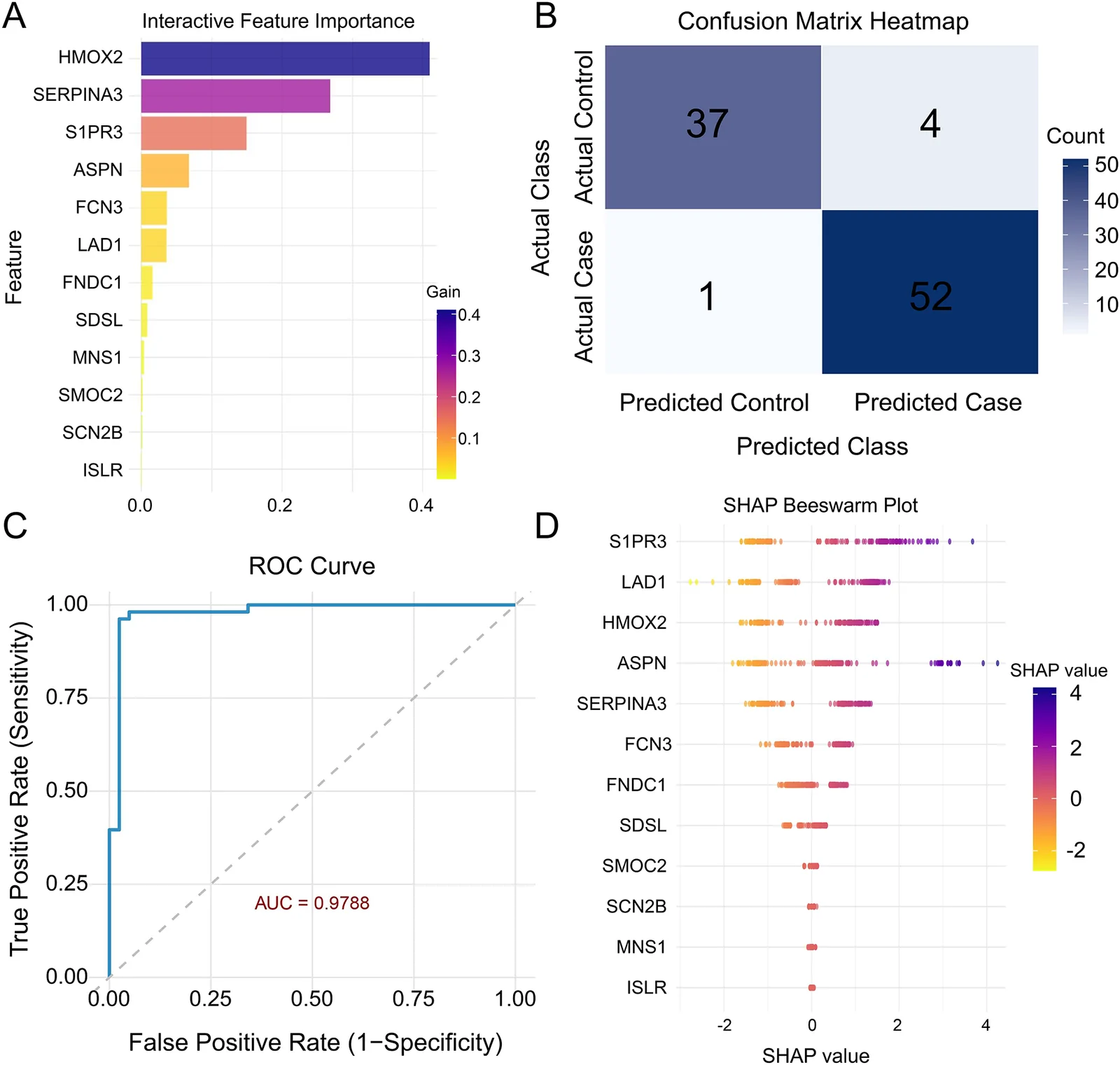
Machine learning-driven biomarker prioritization and experimental validation in vascular aging and heart failure models. A: Feature importance plot derived from the XGBoost model, emphasizing the most significant predictors based on their contribution to outcome prediction. This visual representation helps identify key genes that have the greatest impact on the model’s performance. B: Confusion matrix heatmap for the XGBoost model, illustrating the model’s performance in terms of true positives, false positives, true negatives, and false negatives. C: Bivariate response analysis evaluating the combined effect of the key genes on the risk of VA and HF. D: Global SHAP summary plot, providing an overview of feature contributions across all predictions.

To comprehensively assess the diverse impacts of the 12 core genes on vascular aging in conjunction with heart failure, this study adopted a combined analytical method that merges the XGBoost machine learning algorithm with SHapley Additive exPlanations (SHAP), as depicted in Fig 4. The feature importance analysis based on gain scores (Fig 4A) highlighted HMOX2 as the most crucial predictor, followed by SERPINA3 and S1PR3. For model validation, stratified sampling was utilized to randomly split the cohort into a training set (70%) and an independent test set (30%). The confusion matrix heatmap and ROC curve (Fig 4B and 4C) demonstrated the model’s robust discriminatory power in predicting HF outcomes, achieving an AUC of 0.9788. Additionally, the global SHAP summary plot illustrated the relative contributions of each feature within the predictive model (Fig 4D).

### Analysis of S1PR3, HMOX2, and SERPINA3 expression and cardiac function in doxorubicin-induced models

To further investigate the molecular mechanisms underlying vascular aging and heart failure, we established in vitro and in vivo models using DOX intervention.

**Fig 5:**
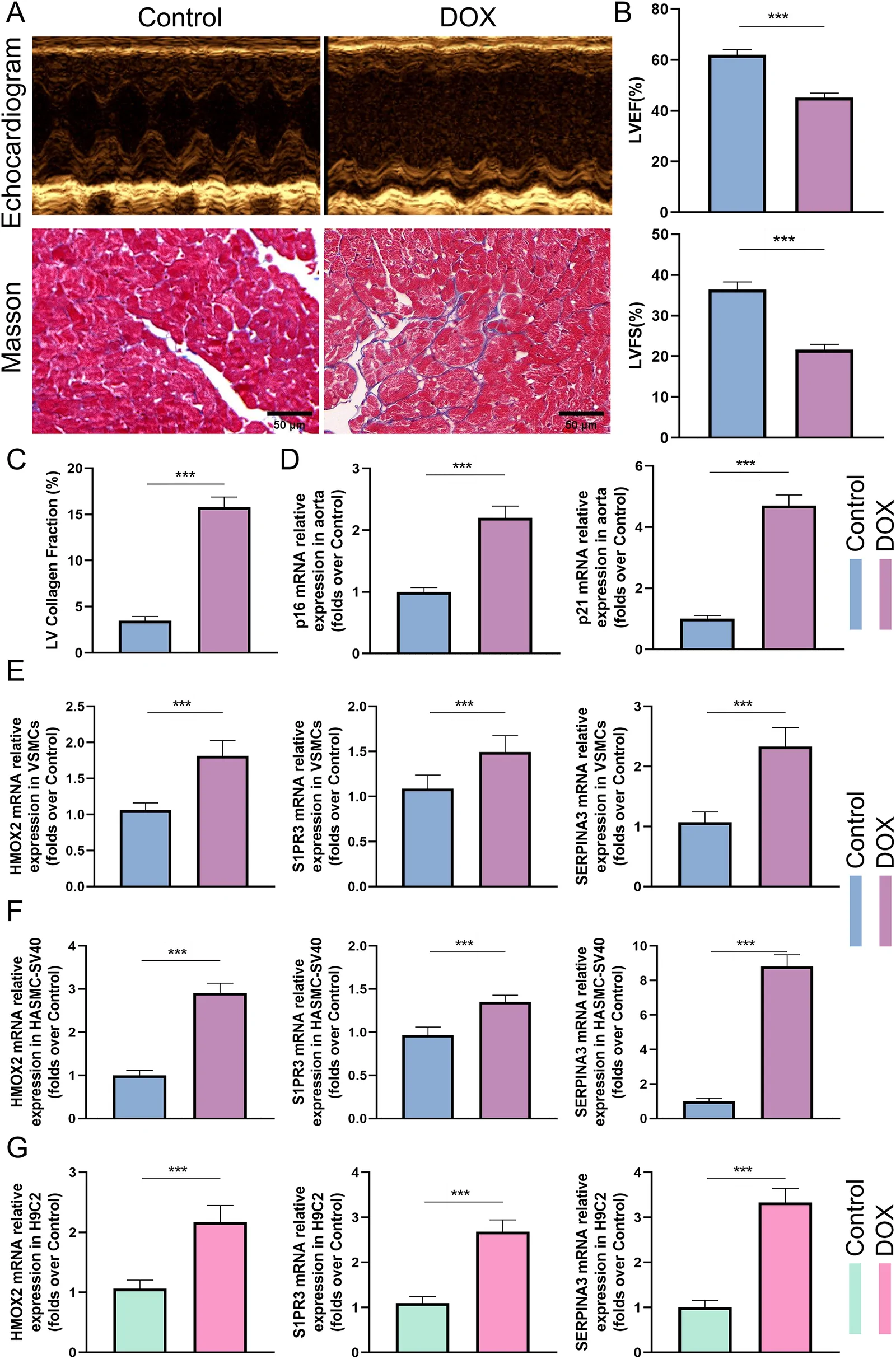
Assessment of cardiac function, myocardial fibrosis, and senescence markers in mice and cell models. A: Representative echocardiographic images and Masson’s trichrome-stained sections of mouse hearts from both groups (n = 5). B, C: Quantitative analyses of (B) ejection fraction (EF) and fractional shortening (FS), and (C) cardiac collagen volume fraction (n = 5). D: Relative mRNA expression levels of P16 and P21 in mouse aortic tissues (n = 5). E-G: Relative mRNA expression of HMOX2, S1PR3, and SERPINA3 in (E) primary vascular smooth muscle cells (VSMCs), (F) immortalized Human Aortic Smooth Muscle Cells (HASMCs), and (G) H9C2 cardiomyocytes under senescence or heart failure conditions (n = 5). Data are presented as mean ± SD.

In the mouse model, administration of DOX significantly induced cardiac dysfunction. Echocardiographic assessment revealed a notable decrease in left ventricular ejection fraction and fractional shortening compared to the saline-treated control group. Consistent with the functional impairment, histological analysis of heart tissue sections using Masson’s trichrome staining demonstrated a substantial increase in collagen deposition, indicating pronounced myocardial fibrosis (Fig 5A-C). Furthermore, qPCR analysis performed on vascular tissues isolated from the same animals showed a significant increase in the mRNA expression levels of the senescence-associated markers P16 and P21. (*P* < 0.05; Fig 5D).

Our in vitro models demonstrated significant transcriptional alterations. In the vascular aging model, both rat primary aortic smooth muscle cells and the HASMC subjected to DOX treatment (1.0 μM, 24 h) followed by a 3-day recovery period showed a marked upregulation in the mRNA expression of key genes (S1PR3, HMOX2, and SERPINA3) compared to PBS-treated controls (*P* < 0.05; Fig 5E, F). Similarly, in the heart failure model using H9C2 cardiomyoblasts, DOX intervention robustly enhanced the transcription of the same gene set (*P* < 0.05; Fig 5G).

**Fig 6:**
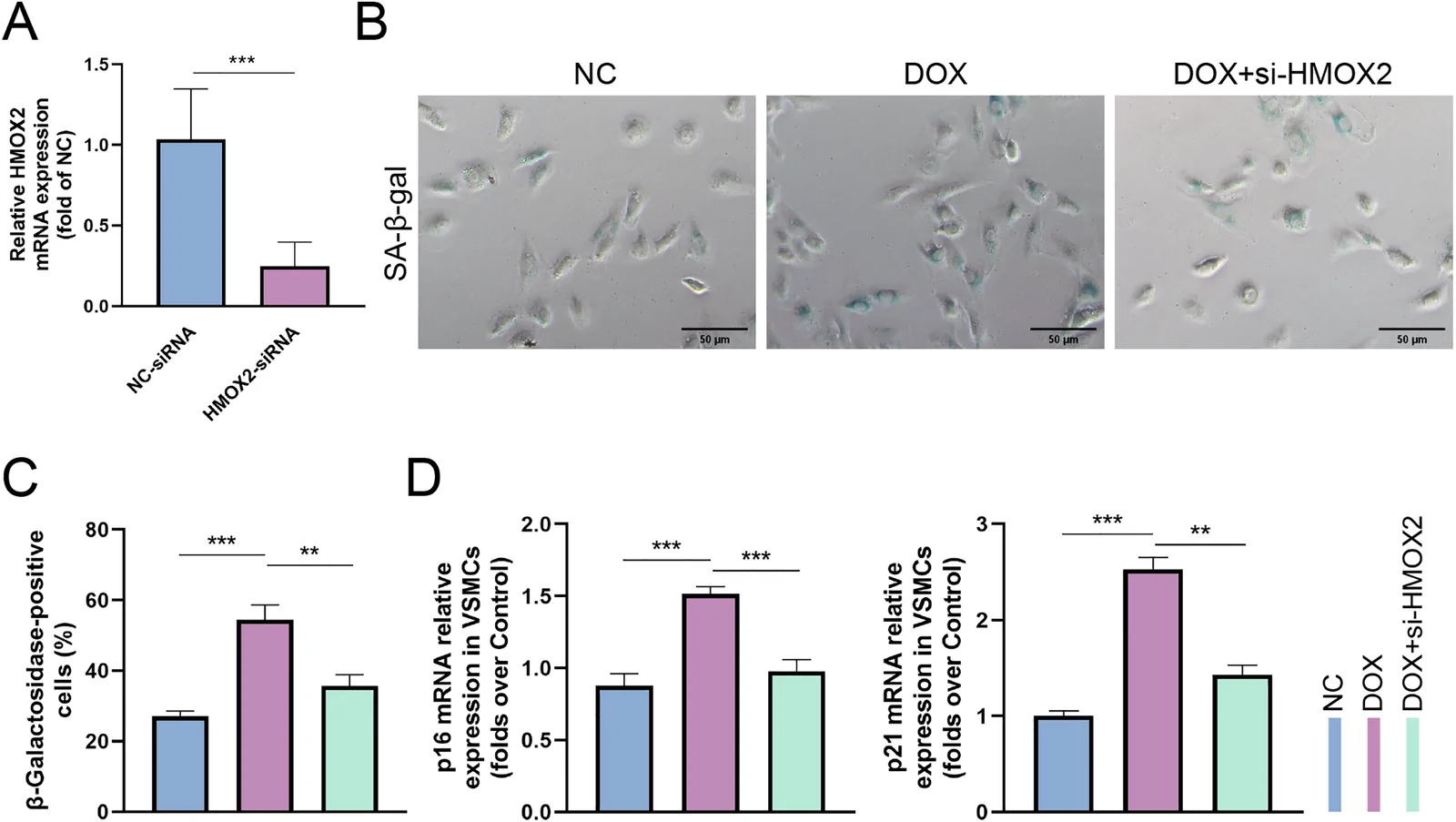
HMOX2 knockdown attenuates DOX-induced senescence in vascular smooth muscle cells. A: RT-qPCR validation of HMOX2 knockdown efficiency in VSMCs transfected with HMOX2-specific siRNA or negative control siRNA (NC-siRNA) (n = 3). B, C: Representative images of SA-β-gal staining and quantification of SA-β-gal-positive VSMCs in the Control, DOX, and DOX + si-HMOX2 groups (scale bar = 50 μm; n = 5). D: Relative mRNA expression levels of P16 and P21 in VSMCs in the Control, DOX, and DOX + si-HMOX2 groups (n = 5). Data are presented as mean ± SD. **P < 0.01, ***P < 0.001.

To further evaluate the functional relevance of HMOX2, we performed siRNA-mediated knockdown in the VSMC senescence model. RT-qPCR confirmed that transfection with HMOX2-specific siRNA significantly reduced HMOX2 mRNA expression compared with NC-siRNA, verifying effective HMOX2 knockdown (Fig 6A). Under DOX-induced stress, HMOX2 knockdown was associated with a significant reduction in SA-β-gal-positive cells (Fig 6B, C) and decreased mRNA expression of the senescence-associated markers P16 and P21 (Fig 6D). These findings indicate that HMOX2 knockdown attenuated the senescence phenotype in DOX-treated VSMCs.

## Discussion

The present study suggests a potential molecular axis linking VA and HF through integrative bioinformatics and explainable ML. By intersecting HF-associated DEGs, vascular aging biomarkers, and co-expression networks, we identified 272 CGs enriched in pathways potentially involved in vascular-cardiac crosstalk. Subsequent ML-driven prioritization highlighted HMOX2 as a potential key player in this pathological interplay.

Mounting evidence positions vascular aging as a precursor to myocardial remodeling, mediated through endothelial dysfunction, oxidative stress, and impaired angiogenesis [4,12–14]. Our functional enrichment analysis of CGs revealed overlaps with these processes, including “regulation of angiogenesis” and “cGMP-PKG signaling”. The latter pathway, known to regulate nitric oxide (NO) -dependent vasodilation, has recently been implicated in age-related arterial stiffening and HF with preserved ejection fraction [15–17]. Notably, the downregulation of cGMP-PKG signaling components in our dataset mirrors observations in aging vasculature. This suggests that reduced vascular NO bioavailability may exacerbate cardiac afterload and diastolic dysfunction and is consistent with findings from preclinical models [18–20]. Furthermore, the enrichment of “cytoskeleton in muscle cells” among CGs underscores the role of VSMC stiffening in HF progression, a phenomenon driven by age-related collagen deposition and titin isoform switching. These findings suggest that vascular aging may play an active role rather than being merely a bystander in myocardial decompensation [4,13].

The identification of HMOX2 as the top-ranked biomarker through SHAP-XGBoost analysis represents a notable finding that warrants careful interpretation regarding its functional directionality. Unlike its widely studied paralog HMOX1, which is robustly induced under oxidative stress, HMOX2 is constitutively expressed and serves as a baseline regulator of heme catabolism, carbon monoxide (CO) production, and intracellular iron homeostasis [21]. A comprehensive review by Ayer et al. highlights that HMOX2 is essential for maintaining vascular endothelial function: Hmox2-deficient mice exhibit reduced eNOS expression, increased endothelial oxidative stress, and elevated inflammatory cytokine production, collectively indicating a protective role under physiological conditions [22]. However, our in vitro data reveal that HMOX2 silencing paradoxically attenuates rather than exacerbates doxorubicin-induced senescence in VSMCs, as demonstrated by decreased SA-β-gal staining and reduced P16/P21 expression (Fig 6). This apparently contradictory finding may reflect the context-dependent dual role of HMOX2 in cellular stress responses. Under sustained pathological stress such as doxorubicin treatment, the constitutive enzymatic activity of HMOX2 may inadvertently contribute to cellular damage through at least two potential mechanisms. First, the continuous degradation of heme by HMOX2 releases ferrous iron (Fe²⁺), which can catalyze Fenton-type reactions to generate highly reactive hydroxyl radicals, promoting lipid peroxidation, DNA damage, and ultimately cellular senescence [23]. Indeed, iron-dependent oxidative injury has been implicated as a driver of senescence in vascular cells, and genetic deletion of Hmox2 has been shown to reduce oxidative stress markers in endothelial cells under certain conditions [22]. Second, while modest CO production exerts cytoprotective effects through cGMP-PKG signaling, excessive or sustained CO generation under severe oxidative stress may impair mitochondrial respiration and enforce cell cycle arrest, paradoxically reinforcing the senescent phenotype [24,25].

Thus, the elevated HMOX2 expression observed in doxorubicin-treated cells may represent an initial compensatory response to heme overload and oxidative stress that, when chronically sustained under pathological conditions, becomes maladaptive by perpetuating iron-catalyzed oxidative damage. Silencing HMOX2 would limit this secondary iron toxicity, thereby partially alleviating the senescence burden. Consistent with this interpretation, Cetin-Atalay et al. demonstrated that germline Hmox2 deletion causes cardiomyopathy and downregulation of cardiac contractility genes, indicating that complete and chronic loss of HMOX2 is detrimental [23], whereas our acute siRNA-mediated knockdown in an in vitro senescence model reveals a distinct, stress-specific aspect of HMOX2 function. These observations collectively suggest that the role of HMOX2 in vascular aging and heart failure is finely tuned by the cellular redox environment and the duration and severity of stress, rather than being uniformly protective or pathogenic. This dual role aligns with the emerging concept that heme oxygenases function as ‘rheostats’ that can shift between beneficial and detrimental outcomes depending on the pathophysiological context.

We acknowledge that our current data do not establish causality, as we have not measured intracellular labile iron concentrations, reactive oxygen species levels, or CO production rates following HMOX2 modulation. Future studies employing iron chelators, CO scavengers, or inducible conditional knockout models are necessary to dissect whether iron-mediated oxidative damage indeed underlies the pro-senescence activity of HMOX2 observed in our doxorubicin model. Additionally, it is important to consider whether HMOX1, the stress-inducible paralog of HMOX2, is upregulated as a compensatory mechanism upon HMOX2 silencing. Although we did not assess HMOX1 expression in this study, such compensation, if present, could partially confound the interpretation of the siRNA phenotype, potentially by providing an alternative source of CO and biliverdin that counterbalances the loss of HMOX2 activity. Conversely, the absence of HMOX1 induction would suggest that the alleviation of senescence following HMOX2 knockdown primarily results from the reduction of iron-mediated oxidative stress. We have therefore added HMOX1 expression profiling to the list of essential follow-up experiments required to fully resolve the respective contributions of these two heme oxygenase isoforms in doxorubicin-induced vascular senescence.

Our machine learning approach demonstrates the value of explainable AI in identifying potential biomarkers. While conventional machine learning models often emphasize predictive accuracy at the expense of interpretability, our SHAP analysis highlighted HMOX2 as a key variable influencing the XGBoost model’s output. This finding was consistent with its central role in the GeneMANIA protein interaction network. These results are in line with recent trends in cardiovascular research that combine machine learning with causal inference, such as AI based phenotyping of heart failure subtypes in the UK Biobank studies [6,26].

Doxorubicin is a well-established agent for modeling both heart failure and vascular aging in experimental systems, as evidenced by prior studies documenting its capacity to induce myocardial damage and premature senescence in vascular cells [27–30]. We acknowledge that doxorubicin represents an acute pharmacological injury model and cannot fully recapitulate the complex, chronic process of physiological aging. However, doxorubicin is widely recognized to induce cellular senescence hallmarks, including telomere dysfunction, DNA damage response, and upregulation of p16/p21, which are core features of aging at the cellular level [31,32]. Therefore, despite its limitations, the doxorubicin model provides a valuable and experimentally tractable platform to interrogate stress-induced senescence pathways relevant to vascular aging and heart failure crosstalk. To experimentally validate these findings, we established cellular models of vascular aging and heart failure using doxorubicin treatment. Quantitative PCR results showed that the expression of S1PR3, HMOX2, and SERPINA3 was significantly increased in both vascular smooth muscle cell and cardiomyoblast models after doxorubicin exposure. This consistent upregulation across different cell types suggests that these genes may function as common molecular nodes linking vascular aging and heart failure. At a functional level, S1PR3 has been associated with both vascular inflammation and cardiac fibrosis. HMOX2, which is involved in cellular stress response, may exhibit altered regulation under sustained pathological conditions. SERPINA3, an acute phase protein, may reflect ongoing tissue remodeling processes common to vascular and cardiac deterioration. Together, these three genes point to potential shared pathways underlying endothelial dysfunction and myocardial impairment. Furthermore, our results showed that HMOX2 knockdown functionally mitigated doxorubicin-induced senescence, suggesting an active role for HMOX2 in this pathway. Nevertheless, our data support association and functional relevance under doxorubicin-induced stress conditions, rather than definitive causality in chronic disease progression.

This study identified shared molecular pathways between vascular aging and heart failure by integrating explainable machine learning with experimental validation. Our approach combined feature selection algorithms with biological network analysis to prioritize key genes, which were subsequently verified in cellular and animal models exposed to doxorubicin. However, several limitations should be acknowledged. First, the reliance on bulk transcriptomic data may obscure cell-type-specific contributions [19,33]. Single-cell or spatial transcriptomics could refine these insights. Second, while our models confirmed alterations in the expression of HMOX2, S1PR3, and SERPINA3, the study lacks functional validation using pharmacological inhibitors, genetic modulations, or rescue experiments, and downstream signaling pathways remain unexplored. Third, the absence of longitudinal clinical data limits our ability to assess HMOX2’s prognostic value. Fourth, the public datasets used in this study did not provide individual age information; therefore, age could not be included as a covariate in our analyses. This precludes adjustment for potential age-related confounding effects and represents an inherent limitation of secondary data analysis. Furthermore, the use of doxorubicin to model vascular aging and heart failure, while practical for inducing senescence-related phenotypes, does not fully capture the chronic, progressive nature of physiological aging. Therefore, future studies using naturally aged animal models are warranted to complement our findings, including exploration of HMOX2 as a therapeutic target via gene-editing technologies and evaluation of its potential as a liquid biopsy marker in HF cohorts. Sixth, the senescence attenuation observed after HMOX2 knockdown was not accompanied by measurements of intracellular labile iron, ROS, or CO levels, and potential compensatory HMOX1 regulation was not assessed. These mechanistic questions, as elaborated in the Discussion, warrant dedicated follow-up studies.

In summary, this study integrates computational biology with experimental models to identify and preliminarily validate HMOX2, S1PR3, and SERPINA3 as potential key nodes linking vascular aging and heart failure. While these findings provide a foundation for understanding shared pathophysiology, future functional studies are essential to confirm their causal roles and therapeutic relevance.

## Supporting information

S1 File Raw R Code. This file contains all author-generated R scripts for data processing, bioinformatics analysis, and explainable machine learning model construction.
(DOCX)

S1 Fig Graphical abstract. (TIF)

## Abbreviations

ANOVA: Analysis Of Variance
AUC: Area Under The Curve
BP: Biological Process
CC: Cellular Component
CGs: Consensus Genes
cGMP-PKG: Cyclic Guanosine Monophosphate-Dependent Protein Kinase
DEGs: Differentially Expressed Genes
DOX: Doxorubicin
EF: Ejection Fraction
FDR: False Discovery Rate
FS: Fractional Shortening
GEO: Gene Expression Omnibus
GO: Gene Ontology
HASMC: Human Aortic Smooth Muscle Cells
HF: Heart Failure
KEGG: Kyoto Encyclopedia Of Genes And Genomes
LASSO: Least Absolute Shrinkage And Selection Operator
MAD: Median Absolute Deviation
MF: Molecular Function
ML: Machine Learning
NO: Nitric Oxide
PBS: Phosphate-Buffered Saline
PPAR: Peroxisome Proliferator-Activated Receptor
qPCR: Quantitative Polymerase Chain Reaction
RF: Random Forest
ROC: Receiver Operating Characteristic
ROS: Reactive Oxygen Species
RT-qPCR: Reverse Transcription Quantitative Pcr
SA-β-gal: Senescence-Associated Beta-Galactosidase
SD: Standard Deviation
SHAP: Shapley Additive Explanations
siRNA: Small Interfering Rna
VA: Vascular Aging
VSMC: Vascular Smooth Muscle Cells
WGCNA: Weighted Gene Co-Expression Network Analysis
XGBoost: Extreme Gradient Boosting
