# Supplementary material for: HMOX2-driven crosstalk between vascular aging and heart failure: A multimodal bioinformatics and explainable machine learning approach with experimental validation
Source: PLoS One. 2026 Sep 15;21(9):e0357886. doi: 10.1371/journal.pone.0357886 (PMC13577404; doi:10.1371/journal.pone.0357886)
Supplement: Supplementary file 1 [file pone.0357886.s001.docx]

library(tidyverse)

library(limma)

setwd("GSE57338")

exp <- read.table("GSE57338_series_matrix.txt",header=T,

sep="\t",dec=".",

comment.char="!",na.strings =c("NA"),fill=T )

plate <- read.table("GPL11532-32230.txt",header=T,

quote="",sep="\t",dec=".",

comment.char="#",na.strings =c("NA"),fill=T )

ID <- data.frame(ID_REF = plate$ID, Gene_Symbol = plate$gene_assignment)

ID$Gene_Symbol <-data.frame(sapply(ID$Gene_Symbol,function(x)unlist(strsplit(x,"///"))[1]),stringsAsFactors=F)[,1]

exp <- as.data.frame(exp)

exp <- merge(exp, ID, by='ID_REF')

exp[, grep("Gene_Symbol", colnames(exp))] <- trimws(exp[, grep("Gene_Symbol", colnames(exp))])

exp[exp == ""] <- NA

exp <- na.omit(exp)

exp1 <- avereps(exp, ID = exp$Gene_Symbol)

exp1 <- as.data.frame(exp1)

rownames(exp1) <- exp1$Gene_Symbol

exp1 <- exp1[,-c(1,ncol(exp1))]

write.csv(exp1, "exp_average.csv")

library(limma)

library(pheatmap)

library(ggplot2)

library(ggrepel)

#inputFile="exp_average.csv"

conFile="s1.txt"

treatFile="s2.txt"

logFCfilter=0.5

adj.P.Val.Filter=0.05

dimnames=list(rownames(exp),colnames(exp))

data=matrix(as.numeric(as.matrix(exp)),nrow=nrow(exp),dimnames=dimnames)

rt=avereps(data)

qx=as.numeric(quantile(rt, c(0, 0.25, 0.5, 0.75, 0.99, 1.0), na.rm=T))

LogC=( (qx[5]>100) || ( (qx[6]-qx[1])>50 && qx[2]>0) )

if(LogC){

rt[rt<0]=0

rt=log2(rt+1)}

data=normalizeBetweenArrays(rt)

sample1=read.table(conFile, header=F, sep="\t", check.names=F)

sample2=read.table(treatFile, header=F, sep="\t", check.names=F)

sampleName1=gsub("^ | $", "", as.vector(sample1[,1]))

sampleName2=gsub("^ | $", "", as.vector(sample2[,1]))

conData=data[,sampleName1]

treatData=data[,sampleName2]

data=cbind(conData,treatData)

conNum=ncol(conData)

treatNum=ncol(treatData)

Type=c(rep("Control",conNum), rep("HF",treatNum))

design <- model.matrix(~0+factor(Type))

colnames(design) <- c("HF","Control")

fit <- lmFit(data,design)

cont.matrix<-makeContrasts(HF-Control,levels=design)

fit2 <- contrasts.fit(fit, cont.matrix)

fit2 <- eBayes(fit2)

allDiff=topTable(fit2, adjust='fdr', number=200000)

#allDiffOut=rbind(id=colnames(allDiff),allDiff)

allDiffOut <- allDiff

write.table(allDiffOut, file="all.txt", sep="\t", quote=F, col.names=T)

Type=c(rep("Control",conNum),rep("HF",treatNum))

outData=rbind(id=paste0(colnames(data),"_",Type),data)

write.table(outData, file="normalize.txt", sep="\t", quote=F, col.names=F)

diffSig=allDiff[with(allDiff, (abs(logFC)>logFCfilter & P.Value < adj.P.Val.Filter )), ]

diffSigOut=rbind(id=colnames(diffSig),diffSig)

write.table(diffSigOut,file="diff.txt",sep="\t",quote=F,col.names=F)

geneNum=100

diffSig=diffSig[order(as.numeric(as.vector(diffSig$logFC))),]

diffGeneName=as.vector(rownames(diffSig))

diffLength=length(diffGeneName)

hmGene=c()

if(diffLength>(2*geneNum)){

hmGene=diffGeneName[c(1:geneNum,(diffLength-geneNum+1):diffLength)]

}else{

hmGene=diffGeneName

}

hmExp=data[hmGene,]

Type=c(rep("Control",conNum),rep("HF",treatNum))

names(Type)=colnames(data)

Type=as.data.frame(Type)

pdf(file="Top-100heatmap.pdf", width=9, height=6.5)

pheatmap(hmExp,

annotation=Type,

color = colorRampPalette(c("blue", "white", "red"))(50),

cluster_cols =F,

show_colnames = F,

scale="row",

fontsize = 7,

fontsize_row=5,

fontsize_col=7)

dev.off()

allDiff$logFC[allDiff$logFC>20]=20

allDiff$logFC[allDiff$logFC< -20]=-20

Significant=ifelse((allDiff$P.Value<adj.P.Val.Filter & abs(allDiff$logFC)>logFCfilter), ifelse(allDiff$logFC>logFCfilter,"Up","Down"), "Not")

geneNum=10

diffSig=diffSig[order(as.numeric(as.vector(diffSig$logFC))),]

diffGeneName=as.vector(rownames(diffSig))

diffLength=length(diffGeneName)

hmGene=c()

if(diffLength>(2*geneNum)){

hmGene=diffGeneName[c(1:geneNum,(diffLength-geneNum+1):diffLength)]

}else{

hmGene=diffGeneName

}

hmExp=data[hmGene,]

hmExp <- as.data.frame(hmExp)

top5.genes <- c(as.character(rownames(hmExp)))

allDiff2 <- allDiff

allDiff2$Label = ""

allDiff2$Gene <- rownames(allDiff2)

allDiff2$Label[match(top5.genes, allDiff2$Gene)] <- top5.genes

p = ggplot(allDiff2, aes(logFC, -log10(adj.P.Val)))+

geom_point(aes(col=Significant))+

scale_color_manual(values=c("#00AFBB","#838B8B","red"))+#"#00AFBB","#838B8B","red"##"green", "black", "red"

labs(title = " ")+

theme(plot.title = element_text(size = 16, hjust = 0.5, face = "bold"))+

geom_hline(yintercept = -log10(0.05), linetype = "dashed") +

geom_vline(xintercept = c(-0.5, 0.5), linetype = "dashed")+

geom_label_repel(data = allDiff2,aes(logFC, -log10(adj.P.Val), label = Label),size = 3)

p=p+theme_bw()

pdf(file="Top10-vol2.pdf", width=10.5, height=6)

print(p)

dev.off()

library(clusterProfiler)

library(org.Hs.eg.db)

library(enrichplot)

library(ggplot2)

library(circlize)

library(RColorBrewer)

library(dplyr)

library(ComplexHeatmap)

pvalueFilter=0.05

adjPvalFilter=0.05

colorSel="p.adjust"

if(adjPvalFilter>0.05){

colorSel="pvalue"

}

setwd("LassoGOKEGG")

rt=read.table("input.txt", header=F, sep="\t", check.names=F)

genes=unique(as.vector(rt[,1]))

entrezIDs=mget(genes, org.Hs.egSYMBOL2EG, ifnotfound=NA)

entrezIDs=as.character(entrezIDs)

rt=cbind(rt, entrezIDs)

rt=rt[rt[,"entrezIDs"]!="NA",]

gene=rt$entrezID

#gene=gsub("c\\(\"(\\d+)\".*", "\\1", gene)

kk=enrichGO(gene=gene, OrgDb=org.Hs.eg.db, pvalueCutoff=1, qvalueCutoff=1, ont="all", readable=T)

GO=as.data.frame(kk)

GO=GO[(GO$pvalue<pvalueFilter & GO$p.adjust<adjPvalFilter),]

write.table(GO, file="GO.txt", sep="\t", quote=F, row.names = F)

pdf(file="GObarplot.pdf", width=8, height=15.8)

bar=barplot(kk, drop=TRUE, showCategory=10, label_format=30, split="ONTOLOGY", color=colorSel) + facet_grid(ONTOLOGY~., scale='free')

print(bar)

dev.off()

pdf(file="GObubble.pdf", width=9, height=16)

bub=dotplot(kk, showCategory=10, orderBy="GeneRatio", label_format=30, split="ONTOLOGY", color=colorSel) + facet_grid(ONTOLOGY~., scale='free')

print(bub)

dev.off()

ontology.col=c("#00AFBB", "#E7B800", "#90EE90")

data=GO[order(GO$pvalue),]

datasig=data[data$pvalue<0.05,,drop=F]

BP = datasig[datasig$ONTOLOGY=="BP",,drop=F]

CC = datasig[datasig$ONTOLOGY=="CC",,drop=F]

MF = datasig[datasig$ONTOLOGY=="MF",,drop=F]

BP = head(BP,6)

CC = head(CC,6)

MF = head(MF,6)

data = rbind(BP,CC,MF)

main.col = ontology.col[as.numeric(as.factor(data$ONTOLOGY))]

BgGene = as.numeric(sapply(strsplit(data$BgRatio,"/"),'[',1))

Gene = as.numeric(sapply(strsplit(data$GeneRatio,'/'),'[',1))

ratio = Gene/BgGene

logpvalue = -log(data$pvalue,10)

logpvalue.col = brewer.pal(n = 8, name = "Reds")

f = colorRamp2(breaks = c(0,2,4,6,8,10,15,20), colors = logpvalue.col)

BgGene.col = f(logpvalue)

df = data.frame(GO=data$ID,start=1,end=max(BgGene))

rownames(df) = df$GO

bed2 = data.frame(GO=data$ID,start=1,end=BgGene,BgGene=BgGene,BgGene.col=BgGene.col)

bed3 = data.frame(GO=data$ID,start=1,end=Gene,BgGene=Gene)

bed4 = data.frame(GO=data$ID,start=1,end=max(BgGene),ratio=ratio,col=main.col)

bed4$ratio = bed4$ratio/max(bed4$ratio)*9.5

pdf("GO.circlize.pdf",width=10,height=10)

par(omi=c(0.1,0.1,0.1,1.5))

circos.par(track.margin=c(0.01,0.01))

circos.genomicInitialize(df,plotType="none")

circos.trackPlotRegion(ylim = c(0, 1), panel.fun = function(x, y) {

sector.index = get.cell.meta.data("sector.index")

xlim = get.cell.meta.data("xlim")

ylim = get.cell.meta.data("ylim")

circos.text(mean(xlim), mean(ylim), sector.index, cex = 0.8, facing = "bending.inside", niceFacing = TRUE)

}, track.height = 0.08, bg.border = NA,bg.col = main.col)

for(si in get.all.sector.index()) {

circos.axis(h = "top", labels.cex = 0.6, sector.index = si,track.index = 1,

major.at=seq(0,max(BgGene),by=100),labels.facing = "clockwise")

}

f = colorRamp2(breaks = c(-1, 0, 1), colors = c("green", "black", "red"))

circos.genomicTrack(bed2, ylim = c(0, 1),track.height = 0.1,bg.border="white",

panel.fun = function(region, value, ...) {

i = getI(...)

circos.genomicRect(region, value, ytop = 0, ybottom = 1, col = value[,2],

border = NA, ...)

circos.genomicText(region, value, y = 0.4, labels = value[,1], adj=0,cex=0.8,...)

})

circos.genomicTrack(bed3, ylim = c(0, 1),track.height = 0.1,bg.border="white",

panel.fun = function(region, value, ...) {

i = getI(...)

circos.genomicRect(region, value, ytop = 0, ybottom = 1, col = '#BA55D3',

border = NA, ...)

circos.genomicText(region, value, y = 0.4, labels = value[,1], cex=0.9,adj=0,...)

})

circos.genomicTrack(bed4, ylim = c(0, 10),track.height = 0.35,bg.border="white",bg.col="grey90",

panel.fun = function(region, value, ...) {

cell.xlim = get.cell.meta.data("cell.xlim")

cell.ylim = get.cell.meta.data("cell.ylim")

for(j in 1:9) {

y = cell.ylim[1] + (cell.ylim[2]-cell.ylim[1])/10*j

circos.lines(cell.xlim, c(y, y), col = "#FFFFFF", lwd = 0.3)

}

circos.genomicRect(region, value, ytop = 0, ybottom = value[,1], col = value[,2],

border = NA, ...)

#circos.genomicText(region, value, y = 0.3, labels = value[,1], ...)

})

circos.clear()

middle.legend = Legend(

labels = c('Number of Genes','Number of Select','Rich Factor(0-1)'),

type="points",pch=c(15,15,17),legend_gp = gpar(col=c('pink','#BA55D3',ontology.col[1])),

title="",nrow=3,size= unit(3, "mm")

)

circle_size = unit(1, "snpc")

draw(middle.legend,x=circle_size*0.42)

main.legend = Legend(

labels = c("Biological Process", "Cellular Component", "Molecular Function"), type="points",pch=15,

legend_gp = gpar(col=ontology.col), title_position = "topcenter",

title = "ONTOLOGY", nrow = 3,size = unit(3, "mm"),grid_height = unit(5, "mm"),

grid_width = unit(5, "mm")

)

logp.legend = Legend(

labels=c('(0,2]','(2,4]','(4,6]','(6,8]','(8,10]','(10,15]','(15,20]','>=20'),

type="points",pch=16,legend_gp=gpar(col=logpvalue.col),title="-log10(Pvalue)",

title_position = "topcenter",grid_height = unit(5, "mm"),grid_width = unit(5, "mm"),

size = unit(3, "mm")

)

lgd = packLegend(main.legend,logp.legend)

circle_size = unit(1, "snpc")

print(circle_size)

draw(lgd, x = circle_size*0.85, y=circle_size*0.55,just = "left")

dev.off()

rm(list = ls())

library("clusterProfiler")

library("org.Hs.eg.db")

library("enrichplot")

library("ggplot2")

pvalueFilter=0.05

adjPvalFilter=0.1

colorSel="p.adjust"

if(adjPvalFilter>0.05){

colorSel="pvalue"

}

setwd("9.CGs-KEGG")

rt=read.table("interGene.txt", header=F, sep="\t", check.names=F)

genes=unique(as.vector(rt[,1]))

entrezIDs=mget(genes, org.Hs.egSYMBOL2EG, ifnotfound=NA)

entrezIDs=as.character(entrezIDs)

rt=cbind(rt, entrezIDs)

rt=rt[rt[,"entrezIDs"]!="NA",]

gene=rt$entrezID

#gene=gsub("c\\(\"(\\d+)\".*", "\\1", gene)

kk <- enrichKEGG(gene=gene, organism="hsa", pvalueCutoff=1, qvalueCutoff=1)

KEGG=as.data.frame(kk)

KEGG$geneID=as.character(sapply(KEGG$geneID,function(x)paste(rt$genes[match(strsplit(x,"/")[[1]],as.character(rt$entrezID))],collapse="/")))

KEGG=KEGG[(KEGG$pvalue<pvalueFilter & KEGG$p.adjust<adjPvalFilter),]

write.table(KEGG, file="KEGG.txt", sep="\t", quote=F, row.names = F)

showNum=30

if(nrow(KEGG)<showNum){

showNum=nrow(KEGG)

}

pdf(file="KEGGbarplot.pdf", width=8, height=7)

barplot(kk, drop=TRUE, showCategory=showNum, label_format=100, color=colorSel)

dev.off()

pdf(file="KEGGbubble.pdf", width=9, height=7)

dotplot(kk, showCategory=showNum, orderBy="GeneRatio", label_format=100, color=colorSel)

dev.off()

rm(list = ls())

library(glmnet)

inputFile="normalize.txt"

geneFile="input.txt"

setwd("LASSO")

data=read.table(inputFile, header=T, sep="\t", check.names=F, row.names=1)

geneRT=read.table(geneFile, header=F, sep="\t", check.names=F)

data=data[as.vector(geneRT[,1]),]

data=t(data)

group=gsub("(.*)\\_(.*)", "\\2", row.names(data))

rt=as.data.frame(data)

rt$Type=ifelse(group=="Control", 0, 1)

x=as.matrix(rt[,1:(ncol(rt)-1)])

y=rt[,"Type"]

fit=glmnet(x, y, family = "binomial", alpha=1)

pdf(file="lasso.pdf",width=6,height=5.5)

plot(fit)

dev.off()

cvfit=cv.glmnet(x, y, family="binomial", alpha=1,type.measure='deviance',nfolds = 10)

pdf(file="cvfit.pdf",width=6,height=5.5)

plot(cvfit)

dev.off()

coef=coef(fit, s = cvfit$lambda.min)

index=which(coef != 0)

lassoGene=row.names(coef)[index]

lassoGene=lassoGene[-1]

write.table(lassoGene, file="lasso.gene.txt", sep="\t", quote=F, row.names=F, col.names=F)

outTab=rt[,c(lassoGene, "Type")]

outTab=cbind(id=row.names(outTab), outTab)

write.table(outTab, file="lasso.geneExp.txt", sep="\t", quote=F, row.names=F)

rm(list = ls())

library(randomForest)

set.seed(123456)

setwd("9.RF")

inputFile="lasso.geneExp.txt"

data=read.table(inputFile, header=T, sep="\t", check.names=F, row.names=1)

#data=t(data)

data <- data[,1:(ncol(data)-1)]

group=gsub("(.*)\\_", "\\7", row.names(data))

set.seed(123456)

inputFile="normalize.txt"

geneFile="input.txt"

data=read.table(inputFile, header=T, sep="\t", check.names=F, row.names=1)

geneRT=read.table(geneFile, header=F, sep="\t", check.names=F)

data=data[as.vector(geneRT[,1]),]

data=t(data)

group=gsub("(.*)\\_(.*)", "\\2", row.names(data))

rt=as.data.frame(data)

rt$Type=ifelse(group=="Control", 0, 1)

data <- rt

#data=t(data)

data <- data[,1:(ncol(data)-1)]

group=gsub("(.*)\\_", "\\7", row.names(data))

rf=randomForest(as.factor(group)~., data=data, ntree=2000)

pdf(file="森林.pdf", width=6, height=6)

plot(rf, main="Random forest", lwd=2)

dev.off()

optionTrees=which.min(rf$err.rate[,1])

optionTrees

rf2=randomForest(as.factor(group)~., data=data, ntree=optionTrees)

importance=importance(x=rf2)

pdf(file="GeneIm.pdf", width=6.2, height=5.8)

varImpPlot(rf2, main="")

dev.off()

rfGenes=importance[order(importance[,"MeanDecreaseGini"], decreasing = TRUE),]

rfGenes=names(rfGenes[rfGenes>1])

#rfGenes=names(rfGenes[1:30])

write.table(rfGenes, file="RFGenes.txt", sep="\t", quote=F, col.names=F, row.names=F)

sigExp=t(data[,rfGenes])

sigExpOut=rbind(ID=colnames(sigExp),sigExp)

write.table(sigExpOut, file="imGeneExp.txt", sep="\t", quote=F, col.names=F)

rm(list = ls())

#BiocManager::install("WGCNA")

setwd("WGCNA")

library(limma)

library(pheatmap)

library(ggplot2)

library(ggrepel)

library(tidyverse)

#inputFile="exp_average.csv"

conFile="s1.txt"

treatFile="s2.txt"

logFCfilter=1

adj.P.Val.Filter=0.05	dimnames=list(rownames(exp),colnames(exp))

data=matrix(as.numeric(as.matrix(exp)),nrow=nrow(exp),dimnames=dimnames)

rt=avereps(data)

qx=as.numeric(quantile(rt, c(0, 0.25, 0.5, 0.75, 0.99, 1.0), na.rm=T))

LogC=( (qx[5]>100) || ( (qx[6]-qx[1])>50 && qx[2]>0) )

if(LogC){

rt[rt<0]=0

rt=log2(rt+1)}

data=normalizeBetweenArrays(rt)

sample1=read.table(conFile, header=F, sep="\t", check.names=F)

sample2=read.table(treatFile, header=F, sep="\t", check.names=F)

sampleName1=gsub("^ | $", "", as.vector(sample1[,1]))

sampleName2=gsub("^ | $", "", as.vector(sample2[,1]))

conData=data[,sampleName1]

treatData=data[,sampleName2]

data=cbind(conData,treatData)

conNum=ncol(conData)

treatNum=ncol(treatData)

dataend <- as.data.frame(data)

group_list <- c(rep("Control",conNum),rep("HF",treatNum))

save(dataend,group_list,file = "step1-outputdata.Rdata")

library(WGCNA)

rm(list = ls())

getwd()

dir.create("./2.data quality")

setwd("2.data quality/")

load("../step1-outputdata.Rdata")

exp <- dataend

exp[1:4,1:4]

data <- exp

keep_data <- data[order(apply(data,1,mad), decreasing = T)[1:5000],]

datTraits <- data.frame(row.names = colnames(data),group=group_list)

datTraits$groupNO= ifelse(datTraits$group=="Control",'1','2')

datTraits<-as.data.frame(datTraits)

datExpr0 <- as.data.frame(t(keep_data))

datExpr0[1:4,1:4]

datTraits[1:4,1:2]

gsg <- goodSamplesGenes(datExpr0,verbose = 3)

gsg$allOK

if (!gsg$allOK){

\# Optionally, print the gene and sample names that were removed:

if (sum(!gsg$goodGenes)>0)

printFlush(paste("Removing genes:", paste(names(datExpr0)[!gsg$goodGenes],

collapse = ", ")));

if (sum(!gsg$goodSamples)>0)

printFlush(paste("Removing samples:",

paste(rownames(datExpr0)[!gsg$goodSamples], collapse = ", "))); # Remove the offending genes and samples from the data:

datExpr0 = datExpr0[gsg$goodSamples, gsg$goodGenes]

}

gsg <- goodSamplesGenes(datExpr0,verbose = 3)

gsg$allOK

sampleTree = hclust(dist(datExpr0), method = "average")

par(cex = 0.6)

par(mar = c(0,4,2,0))

plot(sampleTree)

plot(sampleTree, main = "Sample clustering to detect outliers", sub="", xlab="", cex.lab = 1.5, cex.axis = 1.5, cex.main = 2)

abline(h = 60, col = "red")

clust = cutreeStatic(sampleTree, cutHeight = 60, minSize = 10)

table(clust)

keepSamples = (clust==1)

datExpr0 = datExpr0[keepSamples, ]

dev.off()

sampleTree2 = hclust(dist(datExpr0), method = "average")

plot(sampleTree2)

nGenes = ncol(datExpr0)

nSamples = nrow(datExpr0)

save(datExpr0, nGenes, nSamples,file = "Step01-WGCNA_input.Rda")

enableWGCNAThreads()

powers = c(1:20)

sft = pickSoftThreshold(datExpr0, powerVector = powers, verbose = 5)

par(mfrow = c(1,2))

cex1 = 0.9

plot(sft$fitIndices[,1], -sign(sft$fitIndices[,3])*sft$fitIndices[,2],

xlab="Soft Threshold (power)",ylab="Scale Free Topology Model Fit,signed R^2",type="n",

main = paste("Scale independence"));

text(sft$fitIndices[,1], -sign(sft$fitIndices[,3])*sft$fitIndices[,2],

labels=powers,cex=cex1,col="red");

abline(h=0.9,col="red")

plot(sft$fitIndices[,1], sft$fitIndices[,5],

xlab="Soft Threshold (power)",ylab="Mean Connectivity", type="n",

main = paste("Mean connectivity"))

text(sft$fitIndices[,1], sft$fitIndices[,5], labels=powers, cex=cex1,col="red")

softPower =sft$powerEstimate

#softPower = 16

adjacency = adjacency(datExpr0, power = softPower)

TOM = TOMsimilarity(adjacency)

dissTOM = 1-TOM

save(TOM,file = "TOM.Rda")

geneTree = hclust(as.dist(dissTOM), method = "average");

plot(geneTree, xlab="", sub="", main = "Gene clustering on TOM-based dissimilarity",

labels = FALSE, hang = 0.04)

minModuleSize = 30

dynamicMods = cutreeDynamic(dendro = geneTree, distM = dissTOM,

deepSplit = 2, pamRespectsDendro = FALSE,

minClusterSize = minModuleSize);

table(dynamicMods)

dynamicColors = labels2colors(dynamicMods)

table(dynamicColors)

plotDendroAndColors(geneTree, dynamicColors, "Dynamic Tree Cut",

dendroLabels = FALSE, hang = 0.03,

addGuide = TRUE, guideHang = 0.05,

main = "Gene dendrogram and module colors")

MEList = moduleEigengenes(datExpr0, colors = dynamicColors)

MEs = MEList$eigengenes

MEDiss = 1-cor(MEs);

METree = hclust(as.dist(MEDiss), method = "average")

plot(METree, main = "Clustering of module eigengenes",

xlab = "", sub = "")

MEDissThres = 0.7

abline(h=MEDissThres, col = "red")

merge = mergeCloseModules(datExpr0, dynamicColors, cutHeight = MEDissThres, verbose = 3)

mergedColors = merge$colors

mergedMEs = merge$newMEs

plotDendroAndColors(geneTree, mergedColors,"Dynamic Tree Cut",

dendroLabels = FALSE, hang = 0.03,

addGuide = TRUE, guideHang = 0.05,

main = "Gene dendrogram and module colors")

moduleColors = mergedColors

table(moduleColors)

colorOrder = c("grey", standardColors(50))

moduleLabels = match(moduleColors, colorOrder)-1

MEs = mergedMEs

dev.off()

clinical <- read.table("clinical.txt",sep = "\t",row.names = 1,check.names = F,stringsAsFactors = F,header = T)

clinical <- clinical[rownames(datExpr0),]

identical(rownames(clinical),rownames(datExpr0))

#com <- intersect(rownames(clinical),rownames(datExpr0))

#clinical <- exp[com,]

#datExpr0 <- datExpr0[com,]

#identical(rownames(clinical),rownames(datExpr0))

head(clinical)

datTraits = as.data.frame(do.call(cbind,lapply(clinical, as.numeric)))

rownames(datTraits) = rownames(clinical)

sampleTree2 = hclust(dist(datExpr0), method = "average")

traitColors = numbers2colors(datTraits, signed = FALSE)

plotDendroAndColors(sampleTree2,

traitColors,

groupLabels = names(datTraits),

main = "Sample dendrogram and trait heatmap")

dev.off()

MEs=orderMEs(MEs)

moduleTraitCor=cor(MEs, datTraits, use="p")

write.table(file="Step04-modPhysiological.cor.xls",moduleTraitCor,sep="\t",quote=F)

moduleTraitPvalue=corPvalueStudent(moduleTraitCor, nSamples)

write.table(file="Step04-modPhysiological.p.xls",moduleTraitPvalue,sep="\t",quote=F)

textMatrix=paste(signif(moduleTraitCor,2),"\n(",signif(moduleTraitPvalue,1),")",sep="")

dim(textMatrix)=dim(moduleTraitCor)

labeledHeatmap(Matrix=moduleTraitCor,

xLabels=colnames(datTraits),

yLabels=names(MEs),

ySymbols=names(MEs),

colorLabels=FALSE,

colors=blueWhiteRed(50),

textMatrix=textMatrix,

setStdMargins=FALSE,

cex.text=0.7,

cex.lab=0.7,

zlim=c(-1,1),

main=paste("Module-trait relationships"))

dev.off()

modNames = substring(names(MEs), 3)

geneModuleMembership = as.data.frame(cor(datExpr0, MEs, use = "p"))

a <- geneModuleMembership

a <- a %>% rownames_to_column()

MMPvalue = as.data.frame(corPvalueStudent(as.matrix(geneModuleMembership), nSamples))

names(geneModuleMembership) = paste("MM", modNames, sep="")

names(MMPvalue) = paste("p.MM", modNames, sep="")

traitNames=names(datTraits)

geneTraitSignificance = as.data.frame(cor(datExpr0, datTraits, use = "p"))

GSPvalue = as.data.frame(corPvalueStudent(as.matrix(geneTraitSignificance), nSamples))

names(geneTraitSignificance) = paste("GS.", traitNames, sep="")

names(GSPvalue) = paste("p.GS.", traitNames, sep="")

for (trait in traitNames){

traitColumn=match(trait,traitNames)

for (module in modNames){

column = match(module, modNames)

moduleGenes = moduleColors==module

if (nrow(geneModuleMembership[moduleGenes,]) > 1){

outPdf=paste(trait, "_", module,".pdf",sep="")

pdf(file=outPdf,width=7,height=7)

par(mfrow = c(1,1))

verboseScatterplot(abs(geneModuleMembership[moduleGenes, column]),

abs(geneTraitSignificance[moduleGenes, traitColumn]),

xlab = paste("Module Membership in", module, "module"),

ylab = paste("Gene significance for ",trait),

main = paste("Module membership vs. gene significance\n"),

cex.main = 1.2, cex.lab = 1.2, cex.axis = 1.2, col = module)

abline(v=0.8,h=0.5,col="red")

dev.off()

}

}

}

for (mod in 1:nrow(table(moduleColors)))

{

modules = names(table(moduleColors))[mod]

probes = colnames(datExpr0)

inModule = (moduleColors == modules)

modGenes = probes[inModule]

write.table(modGenes, file =paste0(modules,".txt"),sep="\t",row.names=F,col.names=F,quote=F)

}

library(xgboost)

library(SHAPforxgboost)

library(ggplot2)

library(tidyr)

library(reshape2)

library(pROC)

library(viridis)

library(plotly)

library(ggridges)

df <- read.delim("D:/FirstGEOAS/SHAP/SHAP.txt", row.names = 1)

df$response <- factor(df$response, levels = c("Control", "Case"))

print(levels(df$response))

set.seed(123)

train_index <- sample(nrow(df), 0.7 * nrow(df))

train_data <- df[train_index, ]

test_data <- df[-train_index, ]

dtrain <- xgb.DMatrix(data = data.matrix(train_data[, -ncol(train_data)]),

label = as.numeric(train_data$response) - 1)

dtest <- xgb.DMatrix(data = data.matrix(test_data[, -ncol(test_data)]))

xgb_model <- xgboost(

data = dtrain,

objective = "binary:logistic",

nrounds = 500,

max_depth = 6,

eta = 0.1

)

importance_matrix <- xgb.importance(model = xgb_model)

write.csv(importance_matrix,"XGBoost importance_matrix.csv")

xgb.plot.importance(importance_matrix = importance_matrix)

library(ggplot2)

importance_df <- as.data.frame(importance_matrix)

importance_df$Gain <- as.numeric(importance_df$Gain)

ggplot(importance_df, aes(x = reorder(Feature, Gain), y = Gain, fill = Gain)) +

geom_bar(stat = "identity", alpha = 0.8) +

coord_flip() +

scale_fill_viridis(option = "plasma", direction = -1) +

labs(

title = "Feature Importance",

x = "Feature",

y = "Gain"

) +

theme_minimal() +

theme(

plot.title = element_text(hjust = 0.5, size = 14, face = "bold"),

axis.title = element_text(size = 12),

axis.text = element_text(size = 10),

legend.position = "none"

)

library(plotly)

p <- ggplot(importance_df, aes(x = reorder(Feature, Gain), y = Gain, fill = Gain)) +

geom_bar(stat = "identity", alpha = 0.8) +

coord_flip() +

scale_fill_viridis(option = "plasma", direction = -1) +

labs(

title = "Interactive Feature Importance",

x = "Feature",

y = "Gain"

) +

theme_minimal()

ggplotly(p)

ggplot(importance_df, aes(x = reorder(Feature, Gain), y = Gain, fill = Gain)) +

geom_bar(stat = "identity", alpha = 0.9, color = "black", width = 0.7) +

coord_flip() +

scale_fill_gradient(low = "#f7fbff", high = "#08306b") +

labs(

title = "Feature Importance",

subtitle = "Top Features Ranked by Gain",

x = NULL,

y = "Gain"

) +

theme_minimal() +

theme(

plot.title = element_text(hjust = 0.5, size = 16, face = "bold", color = "#333333"),

plot.subtitle = element_text(hjust = 0.5, size = 12, color = "#666666"),

axis.title.y = element_blank(),

axis.text = element_text(size = 10, color = "#444444"),

panel.grid.major.y = element_blank(),

panel.grid.minor = element_blank(),

panel.border = element_blank()

)

ggplot(importance_df, aes(x = reorder(Feature, Gain), y = Gain, fill = Gain)) +

geom_bar(stat = "identity", alpha = 0.8) +

geom_text(aes(label = sprintf("%.1f%%", Gain * 100)), hjust = -0.2, size = 3, color = "black") +

coord_flip() +

scale_fill_viridis(option = "plasma", direction = -1) +

labs(

title = "Feature Importance with Percentages",

x = "Feature",

y = "Gain"

) +

theme_minimal()

\# 示例：假设 importance_matrix 包含多类别的特征重要性

importance_df$Class <- rep(c("Control", "Case"), each = nrow(importance_df) / 2)

ggplot(importance_df, aes(x = reorder(Feature, Gain), y = Gain, fill = Class)) +

geom_bar(stat = "identity", position = "dodge", alpha = 0.8) +

coord_flip() +

scale_fill_manual(values = c("#440154", "#21918C")) +

labs(

title = "Feature Importance by Class",

x = "Feature",

y = "Gain"

) +

theme_minimal()

pred_prob <- predict(xgb_model, dtest)

pred_label <- as.numeric(pred_prob > 0.5)

test_data$response <- as.numeric(test_data$response) - 1

conf_matrix <- table(test_data$response, pred_label)

print(conf_matrix)

library(ggplot2)

library(reshape2)

conf_matrix_df <- as.data.frame.matrix(conf_matrix)

colnames(conf_matrix_df) <- c("Predicted Control", "Predicted Case")

rownames(conf_matrix_df) <- c("Actual Control", "Actual Case")

conf_matrix_df$Actual <- rownames(conf_matrix_df)

library(reshape2)

conf_matrix_long <- melt(conf_matrix_df, id.vars = "Actual", value.name = "Count")

colnames(conf_matrix_long) <- c("Actual Class", "Predicted Class", "Count")

print(conf_matrix_long)

str(conf_matrix_long)

ggplot(conf_matrix_long, aes(x = `Predicted Class`, y = `Actual Class`, fill = Count)) +

geom_tile(color = "white", size = 0.5) +

geom_text(aes(label = Count), color = "black", size = 5) +

scale_fill_gradient(low = "#f7fbff", high = "#08306b", name = "Count") +

labs(

title = "Confusion Matrix Heatmap",

x = "Predicted Class",

y = "Actual Class"

) +

theme_minimal() +

theme(

plot.title = element_text(hjust = 0.5, size = 16, face = "bold"),

axis.title = element_text(size = 14),

axis.text = element_text(size = 12),

legend.position = "right",

panel.grid = element_blank()

)

accuracy <- sum(diag(conf_matrix)) / sum(conf_matrix)

precision <- conf_matrix[2, 2] / sum(conf_matrix[, 2])

recall <- conf_matrix[2, 2] / sum(conf_matrix[2, ])

f1_score <- 2 * (precision * recall) / (precision + recall)

cat("Accuracy:", round(accuracy, 4), "\n")

cat("Precision:", round(precision, 4), "\n")

cat("Recall:", round(recall, 4), "\n")

cat("F1 Score:", round(f1_score, 4), "\n")

metrics_df <- data.frame(

Metric = c("Accuracy", "Precision", "Recall", "F1 Score"),

Value = c(accuracy, precision, recall, f1_score)

)

library(gridExtra)

bar_plot <- ggplot(metrics_df, aes(x = Metric, y = Value, fill = Metric)) +

geom_bar(stat = "identity", width = 0.6) +

geom_text(aes(label = round(Value, 3)), vjust = -0.5, size = 5, color = "black") +

scale_fill_manual(values = c("#0072B2", "#D55E00", "#009E73", "#CC79A7")) +

labs(

title = "Model Performance Metrics",

x = NULL,

y = "Value"

) +

theme_minimal() +

theme(

plot.title = element_text(hjust = 0.5, size = 16, face = "bold"),

axis.title.y = element_text(size = 14),

axis.text = element_text(size = 12),

legend.position = "none"

)

table_plot <- tableGrob(metrics_df,

theme = ttheme_minimal(

core = list(fg_params = list(fontsize = 12)),

colhead = list(fg_params = list(fontsize = 14))

))

grid.arrange(bar_plot, table_plot, nrow = 2, heights = c(3, 1.5))

\# ggsave("performance_bar_plot.png", bar_plot, width = 8, height = 6, dpi = 300)

roc_obj <- roc(as.numeric(test_data$response) - 1, pred_prob)

auc_value <- auc(roc_obj)

cat("AUC:", round(auc_value, 4), "\n")

ggroc_plot <- ggroc(

roc_obj,

legacy.axes = TRUE,

alpha = 0.8,

color = "#0072B2",

size = 1.2

) +

geom_abline(

slope = 1,

intercept = 0,

linetype = "dashed",

color = "gray",

size = 0.8

) +

labs(

title = "ROC Curve",

x = "False Positive Rate (1-Specificity)",

y = "True Positive Rate (Sensitivity)"

) +

theme_minimal() +

theme(

plot.title = element_text(hjust = 0.5, size = 16, face = "bold"),

axis.title = element_text(size = 14),

axis.text = element_text(size = 12),

panel.grid.major = element_line(color = "gray90", size = 0.5),

panel.grid.minor = element_blank(),

legend.position = "none"

)

print(ggroc_plot)

annotate_auc <- ggroc_plot +

annotate(

"text",

x = 0.5,

y = 0.2,

label = paste("AUC =", round(auc_value, 4)),

size = 5,

color = "darkred"

)

print(annotate_auc)

shap_values <- shap.values(xgb_model, X_train = data.matrix(train_data[, -ncol(train_data)]))

print(shap_values$shap_score)

dim(shap_values$shap_score)

shap_df <- data.frame(shap_values$shap_score)

colnames(shap_df) <- colnames(test_data[, -ncol(test_data)])

shap_df$id <- 1:nrow(shap_df)

shap_long <- pivot_longer(shap_df, cols = -id, names_to = "Feature", values_to = "SHAP")

ggplot(shap_long, aes(x = SHAP, y = reorder(Feature, SHAP), color = SHAP)) +

geom_point(alpha = 0.7, size = 1.5) +

scale_color_viridis(option = "plasma", direction = -1) +

labs(title = "SHAP Beeswarm Plot", x = "SHAP Value", y = "Feature", color = "SHAP Value") +

theme_minimal() +

theme(

plot.title = element_text(hjust = 0.5, size = 14, face = "bold"),

axis.title = element_text(size = 12),

axis.text = element_text(size = 10)

)

ggplot(shap_long, aes(x = SHAP, y = reorder(Feature, abs(SHAP)), color = SHAP)) +

geom_point(alpha = 0.7, size = 1.5) +

scale_color_viridis(option = "plasma", direction = -1) +

labs(title = "SHAP Beeswarm Plot", x = "SHAP Value", y = "Feature", color = "SHAP Value") +

theme_minimal() +

theme(

plot.title = element_text(hjust = 0.5, size = 14, face = "bold"),

axis.title = element_text(size = 12),

axis.text = element_text(size = 10)

)

ggplot(shap_long, aes(x = SHAP, y = reorder(Feature, SHAP))) +

geom_density_ridges(scale = 0.9, alpha = 0.6, rel_min_height = 0.01,

fill = "lightblue", color = "black") +

geom_jitter(alpha = 0.5, size = 0.8, width = 0.1, color = "darkred") +

labs(title = "SHAP Beeswarm Plot with Density", x = "SHAP Value", y = "Feature") +

theme_minimal() +

theme(

plot.title = element_text(size = 14, face = "bold"),

axis.title = element_text(size = 12),

axis.text = element_text(size = 10)

)

p <- ggplot(shap_long, aes(x = SHAP, y = reorder(Feature, SHAP), color = SHAP)) +

geom_point(alpha = 0.7, size = 1.5) +

scale_color_viridis(option = "plasma", direction = -1) +

labs(title = "Interactive SHAP Beeswarm Plot", x = "SHAP Value", y = "Feature", color = "SHAP Value") +

theme_minimal()

ggplotly(p)
